# ﻿Description of *Nilssondytesdiversus* gen. et sp. nov. from Venezuela (Coleoptera, Adephaga, Dytiscidae, Cybistrinae)

**DOI:** 10.3897/zookeys.1210.124121

**Published:** 2024-08-29

**Authors:** Kelly B. Miller, Mariano C. Michat, Nelson Ferreira Jr

**Affiliations:** 1 Department of Biology and Museum of Southwestern Biology, University of New Mexico, Albuquerque, NM 87131-0001, USA University of New Mexico Albuquerque United States of America; 2 Instituto de Biodiversidad y Biología Experimental y Aplicada, CONICET-Universidad de Buenos Aires, Buenos Aires, Argentina Universidad de Buenos Aires Buenos Aires Argentina; 3 Laboratório de Entomologia, Departamento de Zoologia, Instituto de Biologia, Universidade Federal do Rio de Janeiro, Rio de Janeiro, RJ, Brazil Universidade Federal do Rio de Janeiro Rio de Janeiro Brazil

**Keywords:** Diving beetle, phylogeny, South America, taxonomy, water beetle

## Abstract

A new genus, *Nilssondytes***gen. nov.**, is described for a unique new species, *Nilssondytesdiversus***sp. nov.**, from Venezuela. This paper corrects an inadvertent mistake in a paper by the authors (Miller et al. 2024) in which the collection of deposition of the holotype of the species was not indicated making the new species unavailable which, therefore, made the new genus unavailable. A review of the relevant parts of the International Code of Zoological Nomenclature is included. Diagnostic features of the new genus and species are discussed and illustrated.

## Abstract (es)

Un nuevo género, *Nilssondytes***gen. nov.** se describe para la única nueva especie *Nilssondytesdiversus***sp. nov.** de Venezuela. Este artículo corrige un error involuntario en un artículo de los autores (Miller et al. 2024) en el que no se indicaba la colección de depósito del holotipo de la especie, lo que hacía que la nueva especie no estuviera disponible y, por lo tanto, el nuevo género no estuviera disponible. Se incluye una revisión de las partes relevantes del Código Internacional de Nomenclatura Zoológica. Se discuten e ilustran las características diagnósticas del nuevo género y especie.

## ﻿Introduction

In a recent paper revising the classification of the diving beetle (Dytiscidae) subfamily Cybistrinae, a new genus and new species were described, “*Nilssondytesdiversus*” (Miller et al. 2024). However, a statement indicating the name and location of the type depository of the new species was inadvertently not included (Miller et al. 2024). According to Article 16.4.2 of the International Code of Zoological Nomenclature (hereafter, “The Code” (ICZN 1999)):

“[Art. 16.4] Every new specific and subspecific name published after 1999, except a new replacement name (a *nomen novum*), for which the name-bearing type of the nominal taxon it denotes is fixed automatically [Art. 72.7], must be accompanied in the original publication.

[Art. 16.4.2] where the holotype or syntypes are extant specimens, by a statement of intent that they will be (or are) deposited in a collection and a statement indicating the name and location of that collection (see Recommendation 16C).” (ICZN 1999).

Because of lack of a statement indicating, “… name and location of that collection,” it seems objectively clear that the species name was not made available (Miller et al. 2024).

Despite the species name not being made available by Miller et al. (2024), it is possible the genus name was made available. However, according to Article 67.2. of The Code, “Species eligible for type fixation (originally included nominal species)” for nominal genera, the type species must be an available name cited in the original publication (ICZN 1999). The complete, relevant section of the Code reads as follows:

“67.2.1. In the meaning of the Code the “originally included nominal species” *comprise only those included in the newly established nominal genus or subgenus, having been cited in the original publication by an available name* (including citation by an incorrect spelling [Art. 67.6]) of a species or subspecies (see Articles 45.6 and 68.2), or having been cited there as the deliberate application of a previous misidentification (see Articles 11.10, 67.13 and 69.2.4)” [emphasis ours]. (ICZN 1999).

Since the only species name included in the genus, and the one explicitly designated as the type species, was not available, the genus name was also not available (Miller et al. 2024).

This paper seeks to correct the errors introduced by that paper and formally introduce a new genus and new species of Cybistrinae from northern South America. The following largely replicates Miller et al. (2024) in order to correct the lapse in leaving out the type depository of the new species and the ramifications of that oversight. Additional discussions about relationships of this species and genus to others including a phylogenetic analysis, diagnostics, and keys to Neoptropical Cybistrinae are provided by Miller et al. (2024).

## ﻿Materials and methods

Methods for specimen preparation and examination follow Miller et al. (2024).

Specimens of known species of South American Cybistrinae genera were examined from several collections (Miller et al. 2024). Collections including examined specimens of the new species described here (holotype and paratypes) are the following:

**MIZA**Museo del Instituto de Zoología Agrícola Francisco Fernández Yépez, Universidad Central de Venezuela, Maracay, Venezuela (L. Joly).

**MSBA**Museum of Southwestern Biology, Division of Arthropods, University of New Mexico, Albuquerque, NM, USA (K.B. Miller).

**SEMC**Snow Entomological Collection, University of Kansas, Lawrence, KS, USA(A.E.Z. Short).

Measurements follow Miller et al. (2024) and are based on the range of available specimens and/or published values and were taken either using a standard steel ruler (longer measurements) or an ocular scale on a Zeiss Discovery V8 dissecting microscope at 50× magnification (shorter measurements). All examined specimens of the new species were measured. Measurements include: (1) total length (TL); (2) greatest width across elytra (GW); (3) greatest pronotal width (PW); (4) greatest width of the head (HW); (5) distance between the eyes (EW); (6) narrowest width of metaventral wing (MV, Fig. 6); and (7) width across lateral portion of metacoxal (MC, Fig. 6). The ratios TL/GW, HW/EW, and MC/MV were also calculated to provide an indication of overall shape, eye size, and relative sizes of morphological features.

Male and female genitalia were dissected using methods similar to Miller (2001), Miller and Bergsten (2014, 2016), Miller et al. (2007, 2009, 2024). Line drawings were created by sketching the structure using pencil and a drawing tube attached to a Zeiss Discovery V8™ microscope then scanning and digitizing the sketch, “inking”, and editing using Adobe Illustrator™.

## ﻿Taxonomy

### ﻿ Nilssondytes gen. nov.

Taxon classificationAnimaliaColeopteraDytiscidae

4F32ED96-FC7E-52A2-9F60-69CE12DFCA7F

https://zoobank.org/2477607A-7AA0-4DE8-A865-56563D3D29D9

Figs 1–10 , 11

#### Remark.

The following description is reproduced with some modifications from Miller et al. (2024).

#### Type species.

*Nilssondytesdiversus* sp. nov., by current designation.

#### Diagnosis.

**Figures 1–10.**
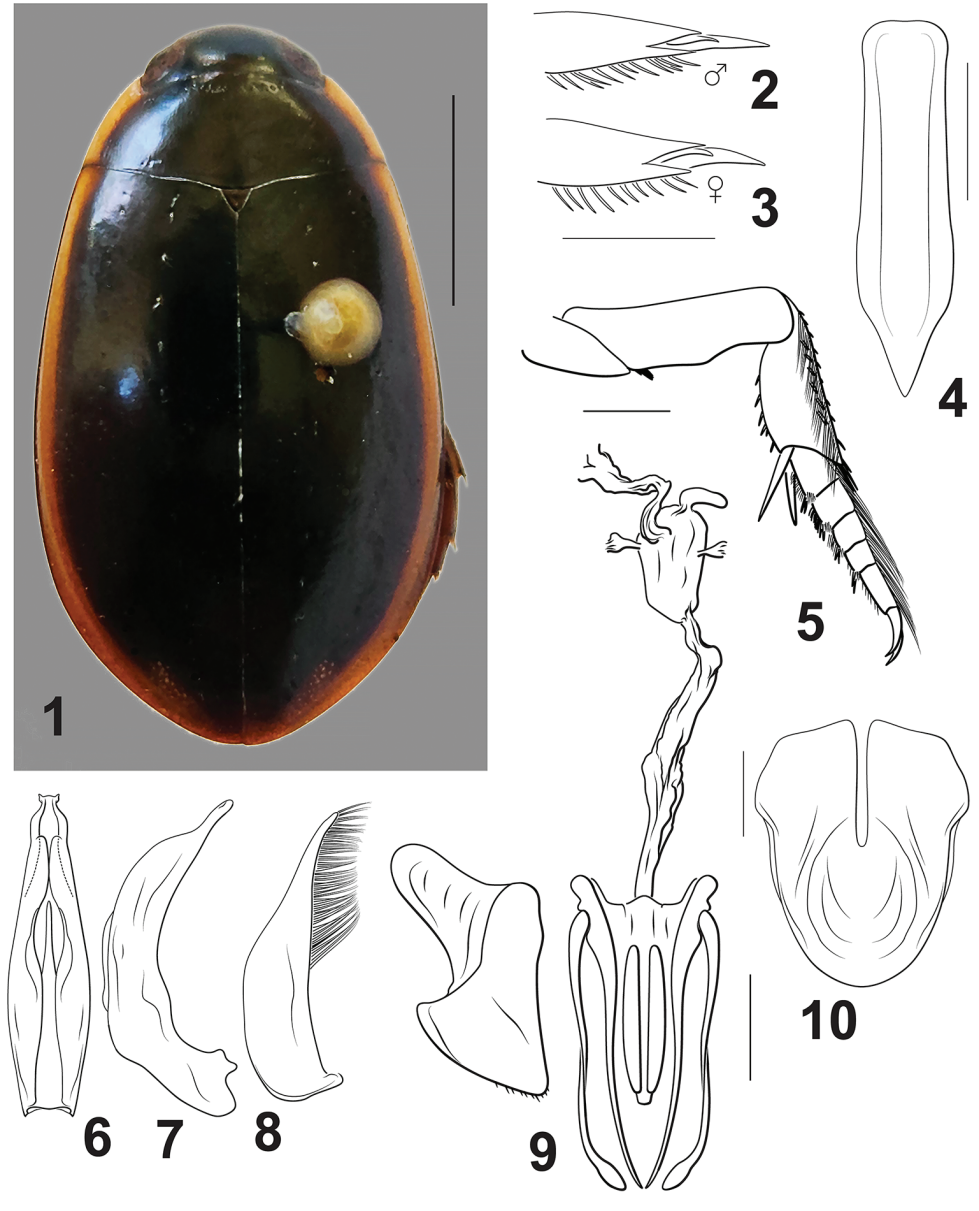
*Nilssondytesdiversus* sp. nov. **1** dorsal habitus **2, 3** metatarsal claws, posterior aspect: **2** male **3** female **4** prosternal process, ventral aspect **5** right mesothoracic leg, posterior aspect **6–8** male genitalia: **6** median lobe, ventral aspect **7** median lobe, right lateral aspect **8** right lateral lobe, right lateral aspect **9** female genitalia (gonocoxae, laterotergites, right gonocoxosternite and internal genitalia), ventral aspect **10** male sternite IX, ventral aspect. Scale bars: 5.0 mm (**1**); 1.0 mm (**2–5, 9, 10**)

From other Cybistrinae this genus differs in having: (1) the metatibial spurs apically simple, (2) metacoxal lines clearly present, (3) the pronotum and elytron with broad, distinct lateral yellow bands along margins (Fig. 1), (4) males and females each with two metatarsal claws, the posterior much reduced in both sexes (Figs 2, 3), (5) the prosternum and prosternal process relatively shallowly but distinctly sulcate (Fig. 4), (6) the medial margins of the male sternite IX straight, not emarginate (Fig. 10), (7) no cluster or line of setae at the apicodorsal angle of the posterior surface of the mesotarsomeres (Fig. 5), and (8) the ventral surface of the metatrochanter with an oblique, transverse groove. The single species in this genus (described below) is somewhat similar in size, shape, and coloration to *Metaxydyteslaevigatus* (Olivier) and may be present among series of that species in collections. *Nilssondytes* differ from *M.laevigatus* in several features (see above) including the presence of yellow lateral elytral margins (Fig. 1) which are absent in *M.laevigatus*. Larvae are unknown.

#### Etymology.

This genus is named *Nilssondytes* from the Greek *dytes* meaning “diver,” and *Nilsson*, after the great diving-beetle worker and excellent friend, Anders N. Nilsson, in honor of his inestimable contribution to the science of diving-beetle biology.

#### Phylogenetic relationships.

The single species of *Nilssondytes* gen. nov. is part of the clade that includes species with an oblique metatrochanteric groove, but it has an unresolved position with respect to other genera (Miller et al. 2024). The presence of a reduced posterior metatarsal claw in both males and females (Figs 2, 3) with straight medial margins of the male abdominal sternite IX (Fig. 10) is a unique combination of features within Cybistrinae. Unique among this larger clade is also the sulcate prosternum and prosternal process (Fig. 4) which is somewhat similar to the Australian genera *Spencerhydrus* Sharp, 1882 and *Sternhydrus* Brinck, 1945.

### ﻿ Nilssondytes diversus sp. nov.

Taxon classificationAnimaliaColeopteraDytiscidae

946585B2-F35C-5E65-B3C0-BCB61748D5D3

https://zoobank.org/EE6E7F8D-5B43-4131-BF61-9692E3F9047E

Figs 1–10 , 11

#### Remark.

The following description is reproduced with some modifications from Miller et al. (2024).

#### Type locality.

Venezuela, Amazonas State, roadside pond ca. 7 km S Samariapo 5°10.900'N, 67°46.078'W, 95 m elev.

#### Diagnosis.

This is the only species in the genus and is characterized by its diagnostic combination (see above). Typically, species-level features include the shape of the male median lobe which is unique. In ventral aspect the apex is abruptly constricted with the apex narrowly truncate with laterally pointed processes (Fig. 6). In lateral aspect, the median lobe is moderately evenly curved on the dorsal margin, apically abruptly narrowed with the apex elongate and slender, apex narrowly rounded (Fig. 7).

#### Description.

***Measurements.***TL = 16.7–19.4 mm, GW = 9.6–10.7 mm, PW = 7.0–8.1 mm, HW = 4.2–4.7 mm, EW = 2.7–2.9 mm, TL/GW = 1.7–1.8, HW/EW = 1.6–1.7, WC/WV = 3.1–3.2. Body shape suboval, slightly expanded posteriorly, widest at ~ 3/5 of length (Fig. 1); lateral margins evenly, continuously curved between pronotum and elytron. Depressed and somewhat flattened in lateral aspect.

***Coloration*** (Fig. 1). Head dark green, anterior clypeal margin yellow, more so laterally, testaceous near eyes. Pronotum dark green with broad lateral yellow margin, posteriorly interrupted and green in three of the four examined specimens, in other specimen yellow extending to posterior angle. Elytron dark green with broad lateral yellow band, separated narrowly from lateral margin, slightly expanded near apex. Ventral surfaces largely black, testaceous on head, basal leg segments and elytral epipleuron.

***Sculpture and structure*.** Head broad, frontoclypeal lines elongate, straight, strongly oblique; anterior clypeal margin broadly, shallowly and evenly concave; dorsal surface evenly covered with fine microsculpture and micropunctures. Pronotum with lateral margins evenly and broadly curved; surface similar to surface of head in microsculpture and micropunctation. Elytral lateral margin evenly and slightly curved for most of length, apically broadly curved; surface of elytron similar to surface of head in microsculpture and micropunctation. Prosternal process apically rounded, ventral surface distinctly sulcate (Fig. 4), apex robust, acutely pointed. Metaventral wing broad, slightly less than 1/3 width of lateral portion of metacoxa (WC/WV = 3.1–3.2); surface smooth, without sculpturing. Lateral portion of metacoxa large, broad, surface smooth, without sculpturing; metacoxal lines short, extending less than half distance across metacoxa. Abdominal ventrites smooth, unsculptured.

***Male genitalia*.** Male median lobe in ventral aspect broad throughout most of length, apically abruptly narrowed, apex laterally produced, submedially with broad, elongate lobes on each side, ventral sclerite short, apically sharp, acuminate, extending to 3/5 length of median lobe, apex sharply pointed (Fig. 6). In lateral aspect shallowly curved, apically abruptly narrowed, apex narrowed, slightly curved, apically narrowly rounded, broad medially (Fig. 7). Lateral lobe broad in basal half, apically distinctly narrowed, apex narrowly rounded, with series of elongate setae along more than apical half of dorsal margin of lateral lobe (Fig. 8).

**Figure 11.**
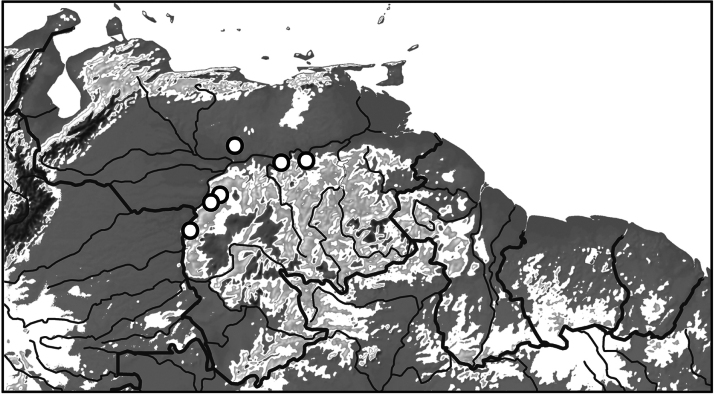
Distribution of *Nilssondytesdiversus* sp. nov. in northern South America.

***Female genitalia*.** With a single genital opening, vagina elongate, slender, with enlarged, bulbous region at base of common oviduct; spermatheca short, curved, at apex of enlarged region, with soft tissue region on each side of enlarged region (Fig. 9); gonocoxae together broad, apically broadly pointed (Fig. 9); gonocoxosternite broad, with elongate anterolateral lobe, with medial margin sublinear, without conspicuous setae (Fig. 9).

***Sexual dimorphism*.** Males have a characteristic broad protarsal palette with ventral adhesive setae. Males also have mesotarsomeres with clumps of posteroventral setae. Females lack pro- and mesotarsal expansions or adhesive setae. Both males and females have two metatarsal claws with the posterior shorter than the anterior (Figs 2, 3), but females have the posterior somewhat more curved than in males (Fig. 3). Females have distinctive microsculpture on the surface of the elytron anteriorly in the form of a field of short striae which is absent in males.

***Variation*.** Five specimens were examined. One specimen has the lateral pronotal yellow band extending to the posterior margin of the pronotum, the others have a narrow dark green separation from the posterior margin.

#### Distribution.

This species is known from few localities in Venezuela along the northwestern margins of the Guiana Shield craton (Fig. 11).

#### Natural history.

The only natural history information available from labels is “roadside pond,” “river margin,” and “rock outcropping.”

#### Etymology.

The species is named from the Latin *diversus*, meaning “different,” in recognition of the different lengths of the metatarsal claws in both males and females (Figs 3, 4).

#### Material examined.

***Holotype***, male deposited in MIZA (see above) labeled, “Venezuela: Amazonas State 5°10.900'N, 67°46.078'W, 95 m ca. 7 km S. Samariapo 15.i.2009; leg. Short, Miller, García, Camacho, Joly VZ09-0115-02X: roadside pond/ SM0846115 KUNHM-ENT [barcode label]/ Holotype: *Nilssondytesdiversus* Miller, Michat & Ferreira-Jr., 2024 [red label with double black line border].” ***Paratypes***, 1 male (SEMC) labeled, “Suapure Venez. Caura River 4.20.1900 [handwritten] E.A. Klages.”, 1 female (MIZA) labeled “Venezuela: Bolivar State 7°41'23.6"N, 64°1'56.0"W, 134 m ca. 14 km E Rio Aro; 5.viii.2008 leg. A. Short $ M. García AS-08-073; rock outcropping/ SM0829328 KUNMH-ENT [barcode label],” 1 female (SEMC) labeled “Venezuela: Guárico State 8°6.226'N, 66°26.228'W, 52m UCV San Nicolasito Field Station: Rio Aguaro; 10.i.2009 leg. Short, Miller, Joly, García, Camacho; VZ09-0110-01A/ SEMC0852602 KUNHM-ENT,” 1 male (MSBA) labeled “Venezuela: Bolivar State 6.58694°N, 67.02912°W Rio Caripito 12.i.2009; leg. Short Miller VZ09-0112-02A: river margin/ SM0844405 KUNHM-ENT [barcode label].” All paratypes with, “…Paratype *Nilssondytesdiversus* Miller, Michat and Ferreira-Jr., 2024 [blue label with black line border].

## Supplementary Material

XML Treatment for Nilssondytes

XML Treatment for Nilssondytes diversus

## ﻿Additional information

### Author ORCIDs

Kelly B. Miller https://orcid.org/0009-0004-1093-4066

Mariano C. Michat https://orcid.org/0000-0002-1962-7976

Nelson Ferreira Jr https://orcid.org/0000-0002-5932-7695

### Data availability

All of the data that support the findings of this study are available in the main text.
